# Long-term consequences of osteoporosis therapy with denosumab

**DOI:** 10.20945/2359-3997000000560

**Published:** 2022-11-10

**Authors:** Francisco Bandeira, Lucian Batista de Oliveira, John P. Bilezikian

**Affiliations:** 1 Universidade de Pernambuco Hospital Agamenon Magalhães Divisão de Endocrinologia e Diabetes Recife PE Brasil Divisão de Endocrinologia e Diabetes, Hospital Agamenon Magalhães, Universidade de Pernambuco (UPE), Faculdade de Ciências Médicas, Recife, PE, Brasil; 2 Columbia University Vagelos College of Physicians and Surgeons Department of Medicine New York NY USA Department of Medicine, Endocrinology Division, Vagelos College of Physicians and Surgeons, Columbia University, New York, NY, USA

**Keywords:** Denosumab, osteoporosis, fractures, osteonecrosis, bone remodeling

## Abstract

Denosumab (DMAb) is a human monoclonal antibody used as an antiresorptive drug in the treatment of osteoporosis. Approval at a dosage of 60 mg every 6 months was based on the results of the randomized, placebo-controlled trial (FREEDOM). The design of this 3-year study included an extension for up to 10 years. Those who were randomized to DMAb continued on drug, while those who were randomized to placebo transitioned to DMAb. The 10-year experience with DMAb provides data on efficacy of drug in terms of reduced fractures and continued increases in bone mineral density (BMD). The 10-year experience with denosumab also provides information about rare complications associated with the use of DMAb, such as osteonecrosis of the jaw (ONJ), and atypical femoral fractures (AFF). This experience provided new insights into the reversibility of effects upon discontinuation without follow-on therapy with another agent. This review focuses upon prolonged treatment with DMAb, with regard to beneficial effects on fracture reduction and safety. Additionally, its use in patients with impaired renal function, compare its results with those of bisphosphonates (BPs), the occurrence/frequency of complications, in addition to the use of different tools, from imaging techniques to histological findings, to evaluate its effects on bone tissue.

## INTRODUCTION

Denosumab (DMAb) is a fully human monoclonal antibody to the receptor activator of nuclear factor-κB (RANKL), inhibiting the development and activity of osteoclasts, followed by supression of bone resorption (1).

DMAb is considered a relevant option in the treatment of osteoporosis owing to the corresponding beneficial outcome of DMAb administration – i.e., reduced risk of fractures (vertebral, non-vertebral, and hip). Rare side effects and risks related to the drug, such as osteonecrosis of the jaw (ONJ), atypical femoral fractures (AFF), and rebound effect should always be considered during clinical management (1,2).

In this narrative review, we address the evidence for the efficacy of DMAb in the treatment of osteoporosis.

## ANTI-FRACTURE EFFICACY

The Fracture Reduction Evaluation of Denosumab in Osteoporosis Every 6 Months (FREEDOM) study, published in 2009, led to the approval of DMAb as a treatment for osteoporosis. This randomized placebo-controlled clinical trial involved 7,808 postmenopausal women with osteoporosis (age, 60-90 years) in which 60 mg of DMAb was administered subcutaneously every six months over a 36-month period. Exclusion criteria were women with other skeletal disorders, current or recent therapies for osteoporosis (use of oral bisphosphonates [BPs] in the last 12 months or for more than 3 years; intravenous BPs, strontium fluoride in the last 5 years; parathyroid hormone or analogues, corticosteroids, estrogen receptor modulator or replacement therapy, calcitonin, and/or calcitriol in the previous 6 weeks), T-Score of ≤ 4.0 in lumbar spine or total femur, with a history of at least one severe or two moderate vertebral fractures, or with serum levels of 25-hydroxyvitamin D < 12 ng/mL. The primary outcome index was the incidence of vertebral fracture after 3 years. Secondary outcomes were non-vertebral and hip fractures. The 36-month incidence of vertebral fractures identified by radiographs in the DMAb group was 2.3% vs. 7.2% in the placebo group, representing a 68% relative risk reduction (p < 0.001) with DMAb use. Similar results were also observed when evaluating the occurrence of multiple vertebral fractures and clinically evident fractures. DMAb also reduced the relative risk of hip fracture by 40% (cumulative incidence of 0.7% in the DMAb group vs. 1.2% in the placebo group; p = 0.04) and non-vertebral fracture by 20% (cumulative incidence of 6.5% in the drug-exposed group vs. 8.0% in the placebo group; p = 0.01). The drug significantly increased lumbar spine and total femur bone mineral density (BMD) (average increases of 9.2% and 6%, respectively), as well as reductions in bone turnover markers (compared to placebo, DMAb provided 86%, 72% and 72% lower serum C-telopeptide of type I collagen [CTx] levels 1 month, 6 months and 3 years after the first administration, respectively, as well as a decrease in procollagen type I N-terminal propeptide [P1NP] levels of 18%, 50% and 76% in the same periods). There was no increase in the incidence of cancer, cardiovascular events, infections, or delay in the healing of fractures associated with DMAb. There was no case of ONJ during the 36-month follow-up (1).

## FREEDOM EXTENSION

Participants who completed the 3-year FREEDOM study, without missing more than one dose of medication, were eligible for a long-term 10-year extension trial. (3–5). Of the 5,928 eligible patients, 4,550 were enrolled for the first 2 years of extension; 2,343 were initially started on DMAb and 2,207 transitioned from placebo to DMAb (crossover group) (3). A total of 1,343 subjects in the long-term treatment group and 1,283 in the crossover group completed the 7-year extension (5).

In this open-label extension trial, DMAb was associated with a greater reduction in the incidence of fractures and continued increases in BMD, when compared to the data from the first 3 years (1,5). The cumulative gain in BMD over the 10 years of continuous DMAb therapy was 21.7% in the lumbar spine. In the total hip, it was 9.2%. Adverse events were rare (seven cases of ONJ in the long-term group and six cases in the crossover group, plus one case of AFF in each group). These data demonstrated the effectiveness and safety of prolonged DMAb treatment (5).

Serum concentrations of CTx and P1NP, prior to the scheduled dose showed, sustained reduction in the long-term group. In the crossover group, there was a rapid decrease in the levels of these bone turnover markers, as seen in the group exposed to DMAb in the first 3 years of FREEDOM, with low levels maintained during the 7-year follow-up (5).

It is worth noting that the proportion of participants who discontinued the study was similar between both groups, as well as the reason for discontinuation, with no greater loss of patients due to adverse events in one of the groups, for example (3–5).

## DENOSUMAB AND KIDNEY FUNCTION

A post hoc analysis from the FREEDOM trial evaluated the safety and efficacy of drug on various levels of renal function. The range was from normal renal function to stage 4 chronic kidney disease (CKD) (estimated glomerular filtration rate of 15-29 mL/min). In all groups, DMAb was not associated with any significant changes in serum calcium or creatinine levels. Effectiveness in reducing fractures was pervasive from CKD stages 1 (n = 842), 2 (n = 4,069) and 3 (n = 2,817). A slight reduction in CKD stage 4 (n = 73) was not significant (6). Since DMAb is not metabolized or excreted by the kidneys (6), these observations are not unexpected. However, a tendency to hypocalcemia, particularly in those with vitamin D deficiency was noted, leading to the recommendation that the serum calcium and 25-hydroxyvitamin D level be checked before administering DMAb (7,8). Patients with stage 5 CKD (on dialysis) were not included in the FREEDOM study. However, a cohort study, which evaluated 121 patients with CKD stage 5 and a control group of 203 patients, concluded that DMAb promoted similar gains in BMD, but there was a higher frequency of hypocalcemia in the group with CKD stage 5. Thus, close monitoring of serum calcium levels in this group of patients is advised (8).

Notably, adynamic bone disease may be present in patients with CKD, a situation in which antiresorptive therapies appeared to have additional side effects and fewer benefits. With the limitation that adynamic bone disease can only be confirmed by bone biopsy, it should be suspected in patients with CKD stage 5 and a serum PTH below 150 pg/mL. If adynamic bone disease in CKD stages 3 or 4 is suspected by serum PTH and bone-specific alkaline phosphatase measurements, a bone biopsy is recommended to confirm the diagnosis (9).

## DENOSUMAB VERSUS BISPHOSPHONATES

BPs are the most used therapeutic agents in the initial treatment of postmenopausal osteoporosis (2,10). Although, like DMAb, the BPs are antiresorptives, their mechanisms of action are remarkably different. By binding to the bone mineral and then becoming ingested by osteoclasts, BPs interfere directly with osteoclastic action. DMAb has no affinity for bone mineral, but rather acts by binding to and inhibiting RANKL, a potent cytokine that stimulates osteoclast differentiation, proliferation, and action (10,11). As an antibody, DMAb circulates throughout the intravascular space (11). The pharmacokinetics of DMAb at 60 mg gives a functional half-life that requires administration every 6 months. If the drug is discontinued, rapid reversibility can lead to a rebound effect, with increases in bone turnover markers, reduction in BMD, and an increase in risk of multiple vertebral fractures (2,10,12).

Different studies have compared the effectiveness of BPs and DMAb in improving bone mass and reducing fractures (10,13,14). A meta-analysis comprised of 10 randomized clinical trials, with a total of 5,361 participants, showed that DMAb more significantly improved BMD in the lumbar spine and hip, at 12 (mean difference: 1.42% at lumbar spine, 1.11% at total hip, and 1.0% at femoral neck; p < 0.001 for all comparisons) and 24 months (mean differences: 1.74% at lumbar spine, 1.22% at total hip, and 1.19% at femoral neck; p < 0.001 for all comparisons), showing superiority in fracture reduction at 24 months (risk ratio 51%, 95% confidence interval [CI] 0.27-0.97). Subgroup analyses showed that in patients previously treated with BPs, the mean difference in BMD improvement with DMAb was more pronounced, suggesting that in those individuals who have had previous exposure to BPs, the use of DMAb provides a greater increase in bone mass than transition to another BP (10). In women previously exposed to oral BPs and intravenous zoledronic acid (ZOL), DMAb was also shown to be superior in improving BMD, and more effective in reducing bone turnover markers (13,14).

## EFFECTS ON BONE MICROARCHITECTURE AND QUALITY

Although BMD is the most used criterion for evaluating bone health and fracture risk, most individuals who sustain a fracture have osteopenia or normal BMD, which reinforces the importance of other factors, like skeletal microstructure, for evaluating bone strength (15). Transiliac bone biopsy can directly assess skeletal microstructure, but it is an invasive and not readily available in clinical practice (15–19). Nevertheless, results from administration of DMAb have been instructive. By bone biopsy, DMAb is associated with normal histology, very low rate of remodeling, an increase in mineralization density, which was more homogeneous (17).

High-resolution peripheral quantitative computed tomography (Hr-pQCT) has also provided important microstructural data after DMAb exposure. It has the capacity to measure volumetric bone density, as well as cortical and cancellous bone microarchitecture (20,21). Of the women included in FREEDOM, 28 exposed to DMAb and 22 from the placebo group underwent HR-pQCT to evaluate cortical porosity in the proximal femur (below the lesser trochanter) (21). Treatment with DMAb reduced cortical porosity, which was associated with improved estimated bone strength and lower levels of CTx (19,21).

More readily available but not as quantitatively reliable, as the bone biopsy and HR-pQCT, is the trabecular bone score (TBS), a software adaptation of lumbar spine Dual-energy X-ray absorptiometry (DXA) (15,16,19,20). TBS estimates differences in the textural homogeneity of the lumbar spine DXA image (15). Utilizing TBS, a retrospective analysis of FREEDOM showed improvement in bone microarchitecture, independent of BMD (22). A greater increase in TBS with DMAb compared to ZOL has also been observed (14). However, the impact of osteoporosis treatment on TBS is less evident than on BMD, which makes this tool less helpful in therapeutic monitoring (15). The role of TBS as a monitoring tool with DMAb treatment needs further investigation.

These imaging modalities, taken together, and followed for up to 10 years, together with the reduction in fracture occurrence over this period of time, suggest that prolonged inhibition of osteoclastic activity by DMAb has overall a beneficial effect on bone quality (5,14,17,19,21).

## RISKS ASSOCIATED WITH DENOSUMAB TREATMENT

ONJ and AFF are rare complications of DMAb. Rapid reversibility of its actions is also a point of discussion.

### Osteonecrosis of the jaw

ONJ is a rare but serious complication of antiresorptive therapy, which can occur with either BPs or DMAb use. It is more commonly observed in patients with neoplasms treated with higher doses than used for osteoporosis (23,24). In the 10-year follow-up of the FREEDOM study and its extension, seven cases of ONJ occurred in the prolonged treatment group and six cases in the crossover group (5). Systematic review pointed out the following differences in imaging tests between ONJ associated with the use of BPs and DMAb. With ONJ associated with BPs, there is an increase in bone sequestration, cortical bone lysis, and an increase in bone density. With DMAb, more bone sequestration, periosteal reactions, and mandibular canal enhancement are observed (23). It should be noted that in addition to the use of drugs, other factors can enhance the risk of ONJ, such as diabetes mellitus, rheumatoid arthritis, systemic arterial hypertension, smoking and poor oral health (dental infection, trauma, or invasive procedures). These other risk factors should always be considered by healthcare professionals, who should advise patients on oral hygiene (24). In patients who develop ONJ during therapy with BPs, 82% had a history of dental procedures prior to the injury (25). Regarding DMAb, analysis derived from the extension of FREEDOM, which evaluated 3,591 patients (78.9% of the 4,550 initially enrolled in the extension), showed that 45.1% underwent at least one invasive oral procedure. From these, 0.68% developed ONJ in comparison with 0.05% in those who did not have a dental procedure (26). The duration of antiresorptive therapy has also been reported as an additional risk factor for ONJ (5,24). However, it should be noted that there is no evidence that discontinuing antiresorptive drugs before dental procedures reduces the risk of ONJ (24).

Epidemiological data on ONJ are difficult to obtain, given its low prevalence, together with limitations in sample size and study design (24). The risk of this complication occurring with DMAb was slightly higher than that observed with ZOL in patients with osteoporosis (0.04% and 0.5% in the 2 and 7-year extensions of FREEDOM, respectively (3,5), vs. 0.017% (27) with ZOL). A real-world study evaluating 9,965 patients registered in the Swiss Society of Rheumatology, of whom 3,068 received BPs, DMAb, or sequential treatment of both, observed 17 cases of ONJ. In 12, ONJ occurred during DMAb treatment (28.3 per 10,000 patients/year), of whom nine were pre-treated with BPs, and five while on BPs only (4.5 per 10,000 patient/year). These results showed a higher risk of ONJ with DMAb compared with BPs (hazard ratio 3.49, 95% CI 1.16-10.47, p = 0.026), with an apparent additional risk in those who used BPs before DMAb (28). A systematic review and meta-analysis that evaluated the risk of ONJ in patients with cancer also showed a higher risk of this complication in patients exposed to DMAb compared to ZOL. However, no differences were observed in terms of prognosis (29).

### Atypical femoral fracture

AFF are typically subtrochanteric and occurring spontaneously or with minimal trauma. They typically involved diaphyseal insufficiency and may have delayed consolidation (2,16,30). In 2013, the American Society of Bone and Mineral Research task force revised the case definition of AFF. To fulfill the definition, the fracture must be located along the femoral shaft, in addition to having at least four of the five main characteristics as follows: associated with minimal or no trauma; fracture line originating in the lateral cortex and substantially transverse (may become oblique when medialization); lateral cortex involvement (only lateral cortex if incomplete, or extending across both cortices if complete); non-comminuted or minimally comminuted; presence of periosteal or endosteal thickening in the lateral cortex (“beaking” or “flaring”). If four of the five main characteristics are present, there is room for clinical judgment (30). AFF were first identified as a potential complication of treatment with BPs, and an association with the use of DMAb was later observed. Although prolonged treatment with BPs leads to a progressive increases in AFF risk, especially beyond five years, there are no data associating the duration of DMAb use with this complication (2,16). A systematic review by the European Calcified Tissue Society found in the literature 22 patients with AFF after DMAb use, of whom 11 were treated for osteoporosis and 11 for metastatic bone disease. Among those treated for osteoporosis, 91% were women and 64% had a history of previous use of BPs (31). From FREEDOM, only two BP-naive patients, out of the 4,550 enrolled in the extension, developed AFF when treated with DMAb, which reinforces the rarity of the complication (5,31). There is no significant evidence that indicates a specific treatment for patients who had AFF; however, there are data that suggest that anabolic drugs, such as teriparatide, can accelerate healing (5,31,32).

An analysis involving FREEDOM data and a hypothetical virtual placebo group demonstrated a satisfactory risk/benefit ratio with 10 years of DMAb therapy, observing 281 and 40 clinical fractures prevented for each episode of AFF and ONJ, respectively (33).

### Reversibility of effect

Another possible complication associated with the use of DMAb is the so-called rebound effect. Its discontinuation, without follow-on use of another anti-osteoporotic drug, leads to an increase in bone turnover and a rapid decrease in BMD, leading to a potentially high risk of multiple vertebral fractures (10,11,34). Multiple fractures occurring within a period of 3 months after delaying a dose of DMAb have been observed (35), which reinforces the need to maintain an anti-osteoporotic therapy after the use of this drug (35–45). There is no ideal regimen established for sequential treatment after DMAb. In view of the wide use of BPs in anti-osteoporotic therapy, this class has already been tested after the use of DMAb in different scenarios (34,36,37). Compared with the bone mass achieved after an average of 2.5 years of DMAb treatment, late infusion of ZOL – 18 months after the last DMAb dose – resulted in a loss of -3.5% in terms of the lumbar spine BMD, whereas early infusion of ZOL – 6 months after the last DMAb administration – generated an additional gain of +1.7% (36). A randomized, open-label and multicenter study, that evaluated the transition from DMAb to alendronate in 115 patients (who received DMAb in year 1 and alendronate in year 2), showed that the majority of subjects maintained or increased their BMD obtained with DMAb, with an average BMD gain above pre-treatment values of 5.9%, 3.6% and 2.5%, for lumbar spine, total femur and femoral neck, respectively. Likewise, at the end of the second year, there was a median reduction of -53.1% in P1NP levels and -54.8% in CTx levels (37). In a retrospective analysis of 797 women exposed to at least two doses of DMAb, it was observed that both prior and subsequent use of BPs protected against the occurrence of fractures after DMAb discontinuation, but the protective effect was stronger when BP was administered after discontinuation. Using BP before DMAb did not further decrease the risk of fractures in subjects who got bisphosphonates after DMAb (34). There is a scarcity of data on the ideal duration of BP therapy after DMAb withdrawal. It is worth noting that case reports have already shown spontaneous multiple vertebral fractures in patients who used alendronate after discontinuation of DMAb (38), a risk that should be emphasized especially in those patients who remain at high or very high risk of fractures after the period of use of DMAb. Despite the limited evidence available, anabolic treatment (especially romosozumab) (39) or a combination (DMAb and teriparatide) (40) appears to be effective in terms of BMD response (39–41). Teriparatide alone does not seem to be the best choice, given the progressive or transient bone loss when this drug is used after DMAb (42). The transition from DMAb to romosozumab (after 12 months of DMAb) appeared to improve lumbar spine BMD and maintain total hip BMD, in addition to possibly preventing the rapid increase in bone turnover marker levels expected after DMAb discontinuation (39).

In conclusion, DMAb is an efficacious agent in the treatment of osteoporosis. Long-term therapy for up to 10 years has demonstrated continued efficacy with an acceptable safety profile for those at continued high risk. As with other therapies, its use requires monitoring with attention to inadvertent withdrawal and to the possibility of rare complications.

**Figure 1:**
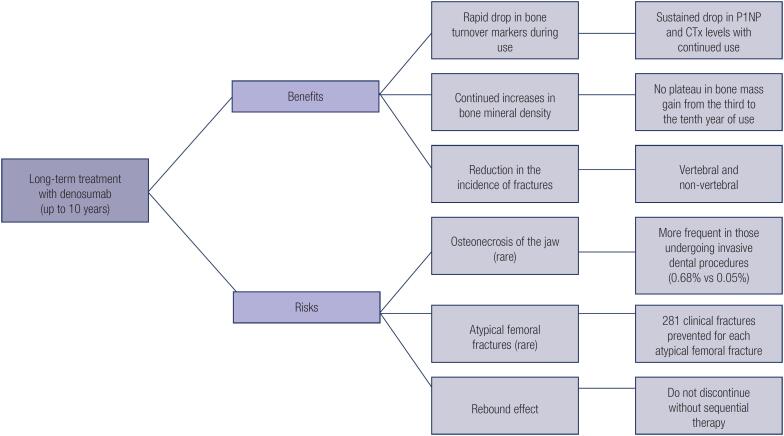
Summary of benefits and risks of long-term treatment with denosumab. CTx: C-telopeptide of type I collagen; P1NP: Procollagen type I N-terminal propeptide.

Figure 1 summarizes the benefits and risks of long-term treatment with denosumab.
